# Enhancing Visible-Light Photocatalytic Activity of AgCl Photocatalyst by CeO_2_ Modification for Degrading Multiple Organic Pollutants

**DOI:** 10.3390/nano15070537

**Published:** 2025-04-01

**Authors:** Li Xu, Ning Yang, Tong Xu, Yang Yang, Yanfei Lv

**Affiliations:** 1 Novel Energy Materials & Catalysis Research Center, Shanwei Innovation Industrial Design & Research Institute, Shanwei 516600, China; bangyoujia@dlmu.edu.cn; 2 Laboratory of Plasma Catalysis, Dalian Maritime University, Dalian 116026, China; yang0717@dlmu.edu.cn (Y.Y.); yanfeilv@dlmu.edu.cn (Y.L.)

**Keywords:** photocatalysis, degradation of pollutant, marine environment, AgCl, CeO_2_

## Abstract

A new type of CeO_2_-modified AgCl catalyst (CeO_2_/AgCl) was prepared by a one-step method, which efficiently inhibits the recombination of photogenerated carriers. During the visible-light degradation process, this catalyst exhibited excellent and stable performance. It could not only effectively degrade rhodamine B (RhB), methyl orange (MO) and crystal violet (CV) but also maintain excellent activity under different environmental conditions. In the RhB degradation experiment in particular, the CeO_2_/AgCl-30 composite with the optimal proportion had a degradation rate 5.43 times that of pure AgCl in the seawater system and 9.17 times that of pure AgCl in the deionized water condition, while also showing excellent stability. Through characterization tests such as XRD, XPS and ESR, its crystal structure, elemental composition and so on were analyzed. Based on the characterization results, the CeO_2_/AgCl composite showed a relatively wide light absorption range and a relatively high photo-induced charge separation efficiency. Meanwhile, it was inferred that the main active species in the reaction process were ·O_2_⁻ and ·OH. Finally, based on its electronic band structure, an S-scheme heterojunction structure was proposed.

## 1. Introduction

As the pace of industrialization quickens, environmental pollution has become increasingly severe. In particular, as a typical source of pollutants, organic pollutants are complex in composition, high in color, highly toxic, and difficult to biodegrade, meaning the massive accumulation of them in water bodies and the air poses a substantial danger to ecosystems and the well-being of human beings. Among them, RhB, CV, MO and other organic dyes, which are widely used in industries such as textiles, printing and dyeing, and leather, are discharged into water bodies in large quantities, causing serious environmental pollution. Traditional sewage treatment methods, such as biological treatment, physical adsorption, and chemical precipitation, often encounter problems when treating organic dye wastewater of this kind, including low treatment efficiency, high cost, or the tendency to generate secondary pollution. Photocatalytic technology, as a highly promising green and environmentally friendly purification method, exhibits great application potential due to its advantages, such as its low energy consumption, mild reaction conditions, and ability to completely mineralize organic pollutants into carbon dioxide and water [1,2].

Photocatalysts can utilize light energy to generate reactive species with strong oxidizing capabilities, such as electron–hole pairs and hydroxyl radicals. These reactive species can entirely degrade organic pollutants, transforming them into non-harmful carbon dioxide and water [3,4]. Among the numerous photocatalytic materials, silver halides (AgX, X = Cl, Br, I) exhibit certain photocatalytic activity because of their distinctive optical and electronic properties [5]. Silver chloride (AgCl) has an appropriate bandgap and can absorb visible light, providing the possibility of solar energy utilization [6,7]. However, single AgCl has some limitations in practical applications. For example, the recombination rate of the carriers generated by light is comparatively elevated, which restricts the photocatalytic efficiency. Its stability is poor, and it is prone to photocorrosion under long-term illumination [8,9,10].

CeO_2_, as an important rare-earth oxide, has excellent oxygen storage and release capabilities, variable valence states, and good electron-transfer characteristics [11,12,13]. Combining an appropriate amount of CeO_2_ with AgCl is expected to establish a novel high-efficiency composite photocatalytic system, effectively curbing the recombination of the carriers generated by light in AgCl and extending the survival time of the carriers, thus augmenting the quantum efficiency in the context of photocatalytic reactions. On the other hand, the structural characteristics of CeO_2_ itself may enhance the stability of composite material and reduce the photocorrosion of AgCl [8,14]. Based on the above background, this study is dedicated to preparing the CeO_2_/AgCl composite material, constructing the S-scheme heterogeneous section and deeply exploring its photocatalytic degradation performance in terms of organic dyes in different environmental systems. It also deeply analyzes the heterojunction mechanism constructed, providing a theoretical basis and technical support for the development of efficient, stable, and practical photocatalytic systems. The aim is to provide new ideas and strategies for the development of efficient and stable photocatalytic materials and promote the further development of photocatalytic technology in the field of environmental purification.

## 2. Experiments

### 2.1. Preparation of CeO_2_

Dissolve 7.2 g of NaOH in 20 milliliters of deionized water. Mark the resulting solution as Solution A and set it aside for later use. Then, pour 2.21 g of Ce(NO_3_)_3_·6H_2_O into 30 milliliters of deionized water. After the solution becomes uniform, slowly add Solution A drop by drop while stirring continuously and then keep stirring for 30 min. Subsequently, subject the mixed solution to a hydrothermal treatment at 100 °C for 24 h. Once the hydrothermal synthesis is completed, wash the product alternately with water and ethanol, then dry it at 100 °C overnight. Finally, place the dried sample in a muffle furnace and calcine it at 600 °C for 2 h to obtain CeO_2_.

### 2.2. Synthesis of CeO_2_/AgCl Photocatalyst

Dissolve 1.18 g of AgNO_3_ in 30 milliliters of deionized water. Subsequently, add a certain amount of CeO_2_ and stir for 1 h. Then, add 3 mL of 36% hydrochloric acid and stir in the dark for 3 h. Finally, wash the obtained sample alternately with water and ethanol, then place it in oven and dry it overnight at 60 °C. Mark the obtained samples as CeO_2_/AgCl. The amounts of CeO_2_ are 0.1 g, 0.3 g and 0.5 g, and corresponding sample names are CeO_2_/AgCl-10, CeO_2_/AgCl-30 and CeO_2_/AgCl-50. AgCl is synthesized following the aforementioned procedure, with the sole exception that CeO_2_ is not added during the process.

The details regarding the characterization, the photocatalytic degradation experiment, and the electrochemical measurement can be found in the Supplementary Information.

## 3. Results and Discussion

**Figure 1:**
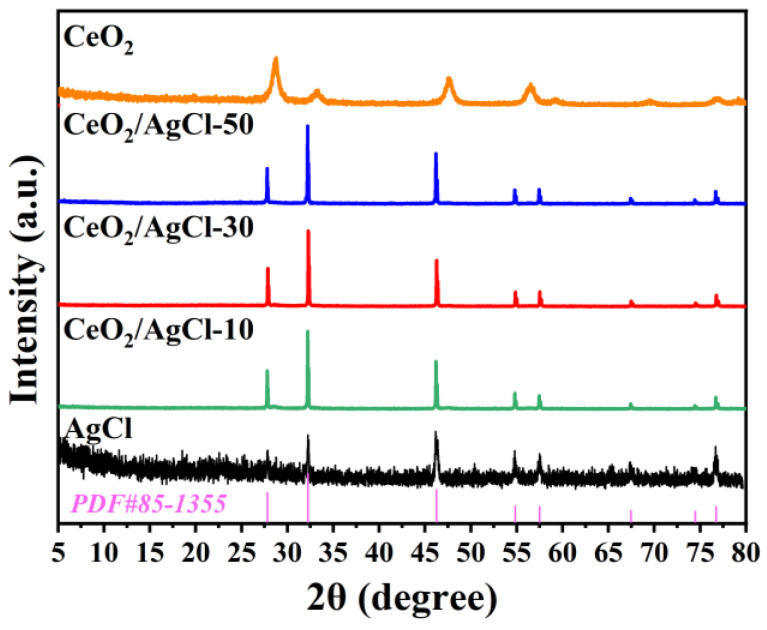
XRD patterns of the AgCl, CeO_2_ and composites with different proportions.

Figure 1 presents the XRD measurements for the CeO_2_, AgCl, and CeO_2_/AgCl samples with different ratios. It is distinctly observable from Figure 1 that both the CeO_2_/AgCl composite materials and AgCl have a relatively narrow full-width at half-maximum, indicating good crystallinity [15]. The diffraction peaks at 27.8°, 32.2°, 46.2°, 54.8°, 57.5°, 67.5°, 74.5°, 76.8° and 85.7° correspond to the (111), (200), (220), (311), (222), (400), (331), (420) and (422) crystal planes of AgCl, which is consistent with the XRD standard card of AgCl (JCPDS No. 85-1355) [16]. Meanwhile, it can be observed that after CeO_2_ is introduced into the material, the composite material still has identical peak positions and diffraction peaks that are comparable to those of AgCl. This observation implies that the crystal structure of AgCl remains stable [17]. The XRD signal of pure AgCl can be observed to have relatively high noise. This may be due to the fact that the particle size of AgCl prepared individually is relatively large, and there may be some potential agglomeration phenomena. At the same time, the FTIR spectra of these materials are shown in Figure S1. It can be observed that compared with AgCl, CeO_2_ exhibits an obvious absorption peak at 500–600 cm^−^¹, which is caused by the vibration of the Ce–O bond [18]. However, in the CeO_2_/AgCl composite, obvious absorption peaks appear at 500–600 cm^−^¹, which further demonstrates the successful introduction of CeO_2_.

**Figure 2:**
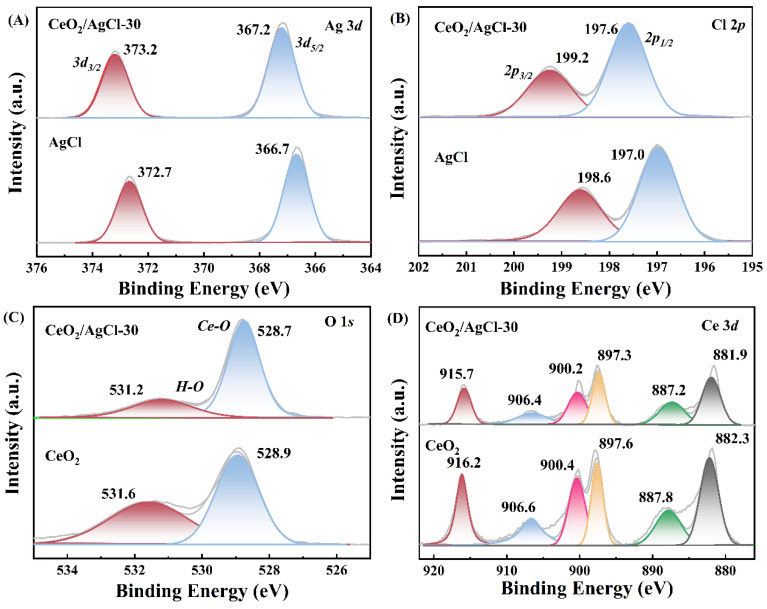
XPS of the AgCl, CeO_2_ and CeO_2_/AgCl-30: (**A**) Ag 3d, (**B**) Cl 2p, (**C**) O 1s, and (**D**) Ce 3d.

To explore the alterations in the chemical states of different elements within the materials, X-ray photoelectron spectroscopy (XPS) analysis was conducted on the AgCl, CeO_2_, and CeO_2_/AgCl-30. Figure 2 analyzes four elements, namely Ag, Cl, O, and Ce. Figure 2A shows the Ag 3d spectrum. The characteristic peaks located at 366.7 eV and 372.7 eV can be identified as the Ag 3d_5/2_ and Ag 3d_3/2_ of Ag^+^ [19]. Similarly, in the CeO_2_/AgCl-30, Ag 3d_5/2_ and Ag 3d_3/2_ correspond to 367.2 eV and 373.2 eV. Figure 2B shows two peaks of Cl^−^ in AgCl, with 197.0 eV corresponding to Cl 2p_1/2_ and 198.6 eV corresponding to Cl 2p_3/2_ [20]. In the CeO_2_/AgCl-30, the binding energies corresponding to Cl 2p_1/2_ and Cl 2p_3/2_ are 197.6 eV and 199.2 eV, respectively. It is worth noting that by comparing the spectra of the above-mentioned AgCl and CeO_2_/AgCl-30, the overall Ag 3d and Cl 2p in the CeO_2_/AgCl-30 shift toward the direction of increased relative binding energy. This is because after CeO_2_ is loaded onto AgCl, the electron cloud density around the Ag^+^ and Cl^−^ decreases, and the attraction of the atomic nucleus to the outermost electrons relatively increases, thus causing the binding energy to shift toward the higher binding energy direction [21,22]. Continuing to observe the O1s spectrum (Figure 2C), the O 1s spectrum is capable of being deconvoluted into two peaks. For CeO_2_, the peak located at 528.9 eV is attributed to the Ce–O bond, while the peak of 531.6 eV originates from surface-adsorbed O [23]. In the CeO_2_/AgCl-30, the corresponding peaks are 528.7 eV and 531.2 eV. In the Ce 3d spectrum (Figure 2D), the peaks at 897.6 eV and 916.2 eV in CeO_2_ can be identified as Ce (IV) 3d_5/2_ and Ce (IV) 3d_3/2_, and the corresponding binding energies in the CeO_2_/AgCl-30 are 897.3 eV and 915.6 eV. Meanwhile, the spectral peaks of Ce (III) 3d_5/2_ and Ce (III) 3d_3/2_ in CeO_2_ are 882.3 eV and 887.8 eV, as well as 900.4 eV and 906.6 eV [24,25], and the corresponding binding energies in the CeO_2_/AgCl-30 are 881.9 eV and 887.2 eV, as well as 900.2 eV and 906.4 eV. By observing the changes in the binding energies of O 1s and Ce 3d in the CeO_2_ and CeO_2_/AgCl-30, it can be found that the binding energies of O 1s and Ce 3d in the CeO_2_/AgCl-30 shift in the direction of relatively decreased binding energy, which echoes the changes in the binding energies from Ag 3d and Cl 2p. Based on the changes in the binding energy observed in the XPS analysis above, it is indicated that an S-scheme heterojunction has formed between the two and chemical bonding exists.

**Figure 3:**
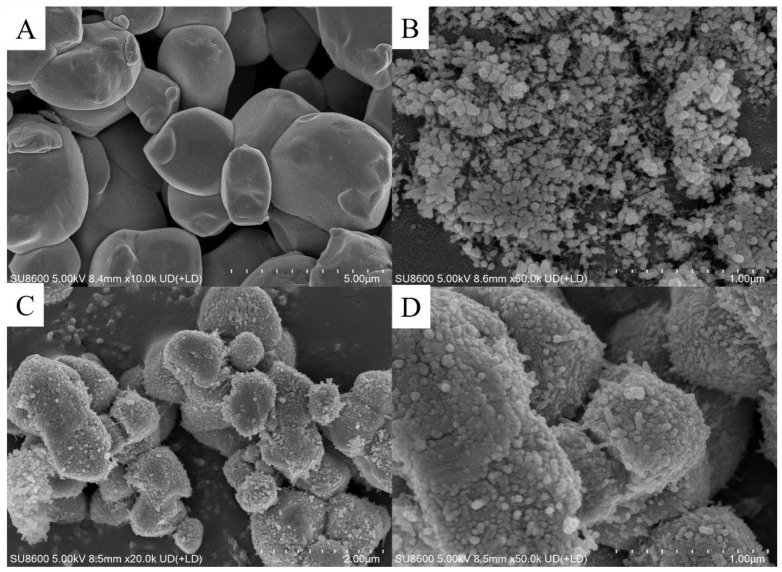
SEM images of (**A**) AgCl, (**B**) CeO_2_ and (**C**), (**D**) CeO_2_/AgCl-30.

The morphologies of the AgCl, CeO_2_ and CeO_2_/AgCl-30 were observed by SEM. As shown in Figure 3A, AgCl appears as relatively large granular particles with a smooth surface. In contrast, in Figure 3B, CeO_2_, as the core, presents nanoparticles with relatively small particle sizes. Through the preparation process for the above-mentioned CeO_2_/AgCl composite material, AgNO_3_ and HCl will take CeO_2_ as the core to generate and encapsulate AgCl. Subsequently, due to the characteristic that AgCl is easily photodecomposed, silver nanoparticles will be formed on the surface of the composite material, as shown in Figure 3C,D. In the composite material CeO_2_/AgCl-30, CeO_2_ and AgCl form a core–shell structure, establishing a close interfacial connection and accompanied by the surface plasmon resonance (SPR) effect of the Ag particles on the surface, which may accelerate the transfer of photogenerated electrons and improve the photocatalytic efficiency of the composite material [26].

Meanwhile, the elemental distribution of the CeO_2_/AgCl-30 was observed through Figure S2, and the response signals of the elements Ag, Cl, O and Ce could be observed. It should be noted that no strong signals of O and Ce were observed from the EDS mapping and elemental distribution diagrams, which further verifies that CeO_2_, as the core of the composite material, is encapsulated by AgCl. The relatively stronger signal of Ag also proves that silver nanoparticles are attached to the surface of the composite material [27].

**Figure 4:**
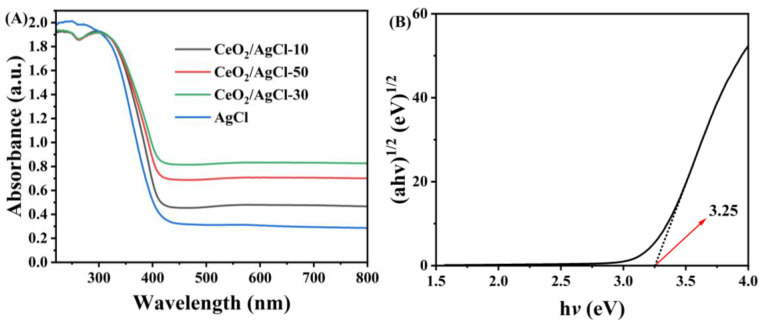
(**A**) UV-vis DRS over AgCl and the composites with different proportions and (**B**) the band gap of AgCl.

The optical traits of the AgCl, CeO_2_, and CeO_2_/AgCl composite materials with different ratios were detected by ultraviolet–visible diffuse reflectance spectroscopy (Figure 4A). Compared to AgCl, the light absorption capacity of the CeO_2_/AgCl composite materials has been remarkably enhanced. An increase in absorption toward longer wavelengths can be observed, which confirms the red-shift phenomenon [28,29]. The red-shift phenomenon observed in the spectra of the nanocomposites confirms the formation of an S-scheme heterojunction between CeO_2_ and AgCl through chemical bonds [30]. Meanwhile, an unstructured tailing phenomenon was observed in the visible-light region, which is attributed to the photodissociation of AgCl into Ag [31,32], as shown in the following equation:

$$2AgCl\overset{hv}{\to }2Ag+{Cl}_{2}$$

It was found that compared with pure AgCl, the composite material exhibited a weak broad absorption band, which is attributed to the surface plasmon resonance response (SPR) of the silver nanoparticles [33,34]. This result further confirms the formation of metallic silver species during the synthesis of the composite material. In Figure 4B, the Eg of AgCl was calculated to be approximately 3.25 eV using the Kubelka–Munk formula [35,36], and the band gaps of CeO_2_ and the composite material CeO_2_/AgCl-30 were also calculated, which were 3.15 eV (Figure S3B) and 3.19 eV (Figure S3C), respectively

**Figure 5:**
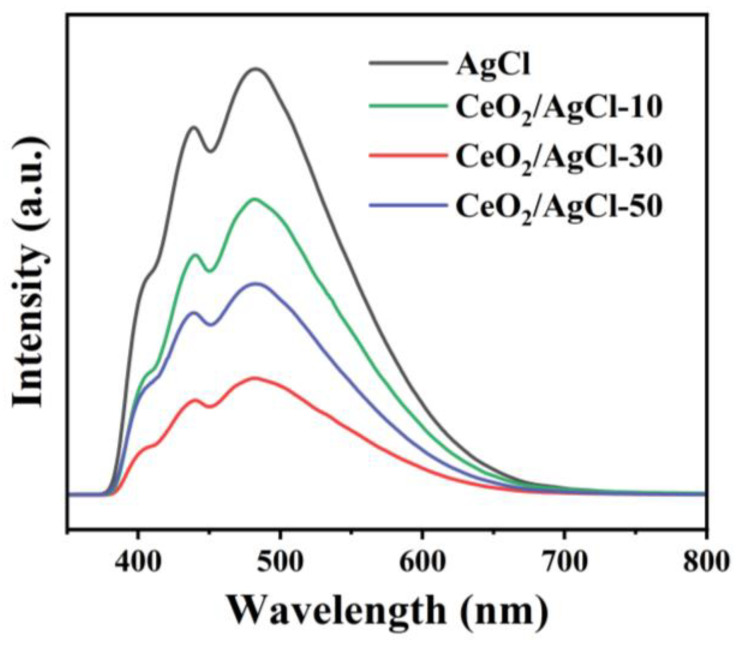
Photoluminescence spectra of AgCl and CeO_2_/AgCl-30.

To probe the charge separation efficiency of pure AgCl and the composites, we tested the PL spectrum to characterize the radiative charge recombination. In Figure 5, two strong absorption peaks can be observed at 481 nm and 435 nm. The peak at 481 nm can be attributed to the PL signal contributed by the Ag^+^-Ag^0^ complex defects during the photodecomposition of AgCl and the SPR effect of the generated Ag^0^ [37]. Meanwhile, the peak at 435 nm may be related to the absorption centers near the Cl^−^ vacancies [38]. For the composite materials with different proportions, the peak intensities all decreased to varying degrees. Among them, the CeO_2_/AgCl-30 exhibited the lowest PL signal, indicating that it had the highest separation efficiency of the e^−^-h^+^ pairs. Specifically, the intensity of the emission peak decreases after CeO_2_ combines with AgCl to form a composite material. This phenomenon can be ascribed to the establishment of an S-scheme heterojunction spanning CeO_2_ and AgCl, which can inhibit the recombination of photogenerated h^+^ and e^−^ [39,40].

**Figure 6:**
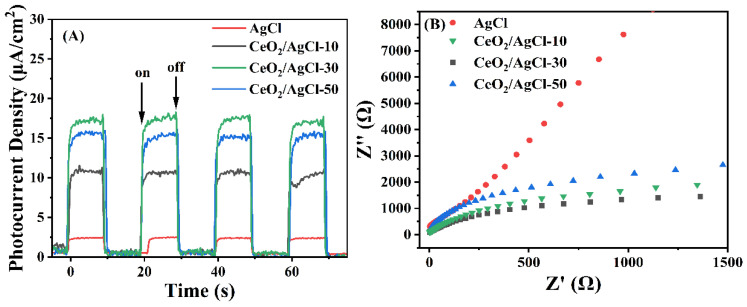
(**A**) Transient photocurrent characteristics and (**B**) electrochemical impedance spectroscopy of AgCl and composites with different proportions.

The photocurrent density can directly reflect the transfer characteristics of photogenerated electrons. The higher the photocurrent density, the higher the separation efficiency of photogenerated carriers [41]. The magnitudes of the transient photocurrents in pure AgCl as well as in the CeO_2_/AgCl composite materials with different ratios during the light switch cycle were determined for testing and the outcomes are presented in Figure 6A. All of the samples produced a photocurrent when exposed to visible-light irradiation, which suggests that they are capable of generating electrons and holes. In contrast, the CeO_2_/AgCl-30 showed the highest photocurrent intensity, demonstrating that it has the best separation efficiency of the photogenerated electron–hole pairs. It is worth noting that it can be observed that after the light is turned on, the photocurrent of the composite material shows a continuous upward trend. This may be attributed to the dynamic process in which AgCl continuously decomposes into Ag nanoparticles under light irradiation. The newly generated Ag continuously, due to its SPR effect, forms a more efficient charge transfer, resulting in the continuous increase of the photocurrent [42]. In addition, with the increase in the proportion, a large amount of agglomeration will occur in the composite material, leading to a decrease in Ag species. This will impede the transfer of electrons and weaken the SPR effect. Therefore, the photocurrent intensity of the sample CeO_2_/AgCl-50 decreases significantly. To further study the charge transfer ability of the samples in visible-light irradiation, the electrochemical impedance spectroscopy (EIS) was recorded. It is widely recognized that a smaller arc radius implies a higher charge separation efficiency [43,44]. In Figure 6B, the order of the arc radius sizes from the samples is as follows: AgCl > CeO_2_/AgCl-10 > CeO_2_/AgCl-50 > CeO_2_/AgCl-30. Apparently, the CeO_2_/AgCl-30 sample exhibits the smallest arc radius, meaning it has a minimum resistance to charge transfer, which further indicates the formation of an S-scheme heterojunction between CeO_2_ and AgCl, which promotes electron migration and corresponds to the XPS results presented in this article. A lower resistance implies that the sample has a better transfer efficiency in terms of the photogenerated electron–hole pairs, which also verifies the statement about the above-mentioned SPR effect. The EIS diagrams of the best samples, CeO_2_/AgCl-30 and AgCl, were fitted to suitable equivalent circuit diagrams through Zview, and the data are shown in Table S1. The proposed equivalent circuit model (Figure S4) consists of four series components: a constant resistor Rs and two resistors R1 and R2 connected in parallel with the constant phase elements (CPE1, CPE2). The results show that the charge transfer resistance of the CeO_2_/AgCl-30 is significantly lower than that of the AgCl, indicating that its electrochemical performance has been significantly improved. It can effectively separate e^−^–h^+^ pairs under light irradiation and promote charge transfer.

**Figure 7:**
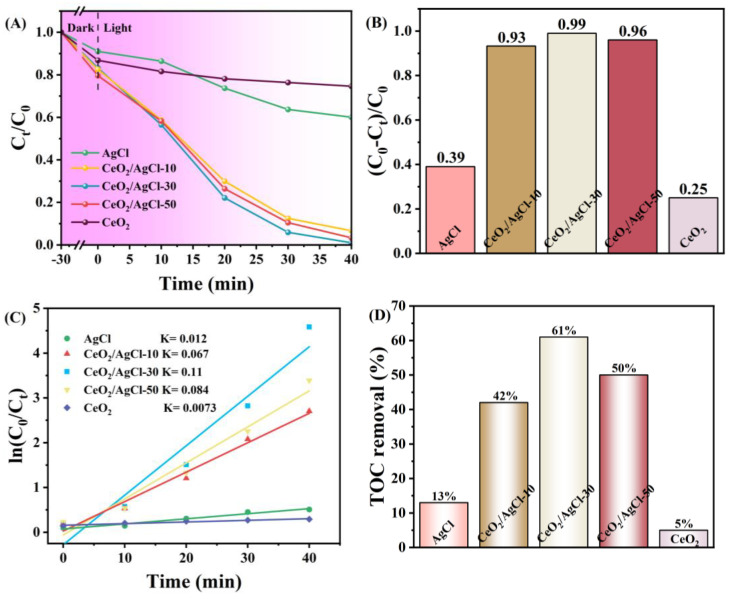
(**A**) Photodegradation curves of RhB under visible light, (**B**) degradation rate, (**C**) first-order kinetic curves of AgCl, CeO_2_ and composites with different proportions and (**D**) TOC removal of the dye solution over different photocatalysts after an irradiation time of 60 min.

The photocatalytic performance of the AgCl and CeO_2_/AgCl composite materials was investigated through the degradation of RhB. Under acidic conditions, a large number of hydrogen ions (H^+^) in the solution will bind to basic sites such as the nitrogen atoms in rhodamine B molecules, causing them to protonate. Protonated rhodamine B carries a positive charge, increasing its ionization degree and making it more soluble in water. As the pH value increases and the alkalinity increases, the concentration of hydroxide ions (OH ^−^) in the solution increases, and the rhodamine B molecules will gradually lose their protons, resulting in a decrease in the ionization degree. To reduce the experimental interference, we used neutral deionized water to prepare the RhB solution. In Figure 7A, the photocatalytic degradation performance of the pure AgCl, CeO_2_, and CeO_2_/AgCl composite materials with different ratios was tested in RhB solution with a concentration of 10 mg/L. It can be observed that pure CeO_2_ has relatively weak degradation performance under these conditions, and AgCl also does not exhibit high photocatalytic activity. However, after introducing CeO_2_ into AgCl, the photocatalytic performance of the composite materials has been significantly improved. Meanwhile, combined with Figure 7B, it can be observed that the degradation rates of the three composite materials have increased significantly. Among them, the composite material CeO_2_/AgCl-30 with a CeO_2_ loading of 30% has the best degradation rate, reaching 99%. It is worthy of note that the optimal degradation rate did not attain 100%. This could be attributed to the presence of a certain degree of error in the experimental apparatus. Additionally, a certain adsorption–desorption dynamic equilibrium might exist between the material and the dye. During the entire decomposition process of the RhB probe molecules, superoxide radicals first attack the conjugated chromophore structure of the RhB molecules, such as adding carbon–carbon double bonds to the RhB molecules, triggering ring-opening reactions. This process disrupts its color structure, causing the color of RhB solution to gradually fade. Subsequently, hydroxyl radicals with strong oxidizing properties and no selectivity begin to take effect. They can attack other chemical bonds in the RhB molecules, such as removing hydrogen atoms from the alkyl chain to form alkyl groups, which are then further oxidized to gradually oxidize the RhB molecules into small organic acids, aldehydes, and other intermediate products. Meanwhile, voids also oxidize the intermediate products that have already been produced, promoting their mineralization. As the reaction continues, these intermediate products are eventually completely mineralized into small molecules such as carbon dioxide CO_2_ and H_2_O that are harmless to the environment. Subsequently, Figure 7C illustrates the first-order kinetic model applicable to the materials and calculates the rate constant K [45]. The Ks the for AgCl, CeO_2_/AgCl-10, CeO_2_/AgCl-30, CeO_2_/AgCl-50, and CeO_2_ are 0.012, 0.067, 0.11, 0.084, and 0.0073 min^−^¹, respectively. The K value for the CeO_2_/AgCl-30 is the uppermost, being 9.17 times that of pure AgCl. It is worth noting that UV-Vis spectroscopy can be employed to study the decolorization of organic dye solutions. However, due to the generation of numerous by-products during the photocatalytic degradation process, it cannot conclusively prove that the dyes have been degraded. In contrast, total organic carbon (TOC) analysis serves as an effective method for evaluating the degradation degree of organic dyes. Figure 7D illustrates the TOC removal rates of RhB after 60 min of light irradiation under the action of different photocatalysts. It can be observed that for the five samples of AgCl, CeO_2_, CeO_2_/AgCl-10, CeO_2_/AgCl-30, and CeO_2_/AgCl-50, the TOC removal rates after 60 min of light irradiation are 13%, 5%, 42%, 61%, and 50%, respectively. This result further confirms that the synthesized CeO_2_/AgCl-30 composite material is a highly efficient photocatalyst for the degradation of organic dyes.

**Figure 8:**
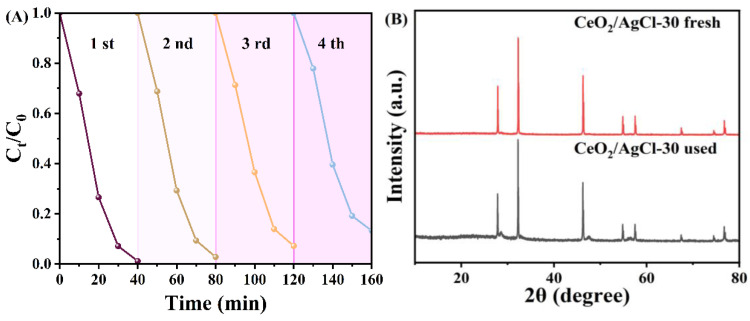

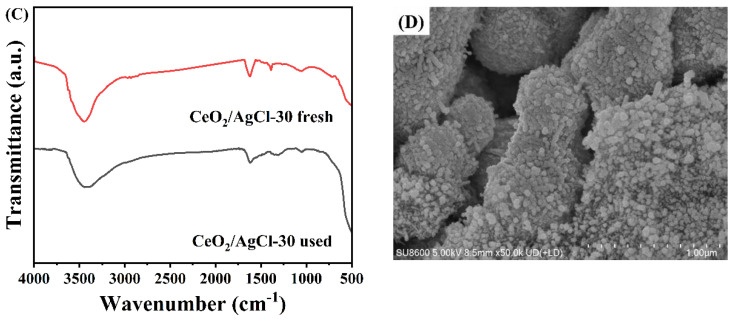
(**A**) Cycling experiments for the photodegradation of RhB using CeO_2_/AgCl-30, (**B**) XRD patterns, (**C**) FTIR of the CeO_2_/AgCl-30 composite before and after photodegradation of RhB, and (**D**) SEM image used for CeO_2_/AgCl-30.

To test the stability of the sample performance, four degradation cycle tests were carried out on the CeO_2_/AgCl-30 material, as shown in Figure 8A. After four cycles of degradation tests, the photocatalytic degradation performance of the material decreased slightly, but the performance was still relatively excellent. Subsequently, XRD and FTIR tests were carried out on the cycled samples. It was found that compared with the fresh samples before the reaction, there was no significant change in their signals. At the same time, combined with the SEM of the cycled samples in Figure 8D, it can be found that the chemical structure and surface characteristics of the cycled samples did not change significantly.

**Figure 9:**
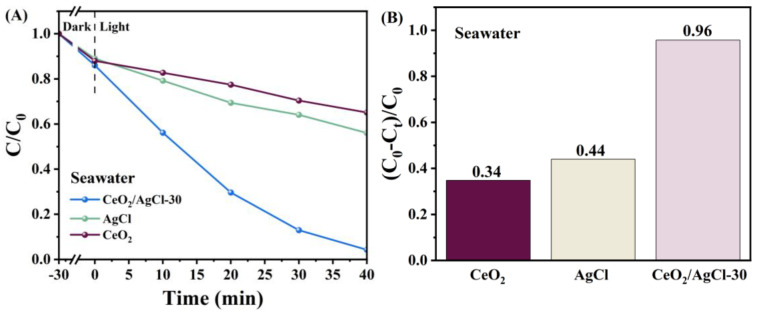
(**A**) Photodegradation curves and (**B**) degradation rate of RhB under visible light in the seawater environment.

To verify whether the CeO_2_/AgCl composite prepared in this work can maintain excellent performance under different environments, a 10 mg/L RhB solution was prepared using seawater to simulate the marine environment. The CeO_2_/AgCl-30 was selected as a representative sample for photocatalytic degradation. Similarly, as can be observed in Figure 9A, compared with CeO_2_ and AgCl, the CeO_2_/AgCl-30 composite still exhibits excellent degradation performance. By observing Figure 9B, it can be known that the degradation rate of the CeO_2_/AgCl-30 can reach 96%. Judging from the above results, it can be demonstrated that the CeO_2_/AgCl catalyst can degrade pollutants in different environments and maintain excellent degradation performance simultaneously.

**Figure 10:**
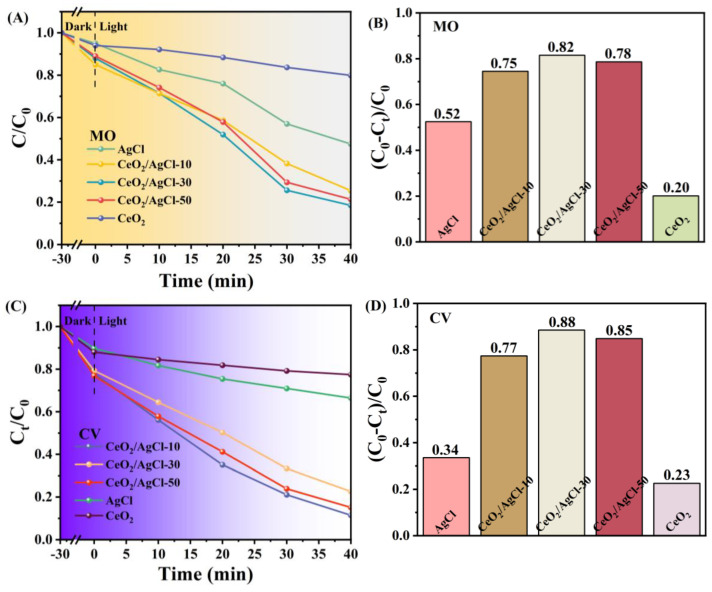
(**A**) Degradation curves, (**B**) degradation rate in MO, (**C**) degradation curves, and (**D**) degradation rate in CV over AgCl, CeO_2_ and composites with different proportions.

To determine whether the CeO_2_/AgCl composite exhibits photocatalytic degradation performance toward other pollutants, the photocatalytic degradation properties of the AgCl, CeO_2_, and the CeO_2_/AgCl composite on methyl orange (Figure 10A,B) and crystal violet (Figure 10C,D) were also investigated. Under identical conditions, the CeO_2_/AgCl-30 continued to exhibit outstanding photocatalytic degradation performance. It can be clearly observed in Figure 10A,C that the performance of the CeO_2_/AgCl-30 was still significantly superior to that of the AgCl. Moreover, the degradation rates of the CeO_2_/AgCl-30 for methyl orange (Figure 10B) and crystal violet (Figure 10D) reached 82% and 88%, respectively. Comparing the degradation effects of the samples on the above-mentioned simulated pollutants indicates that the CeO_2_/AgCl-30 composite can maintain excellent performance for different pollutants and holds great promise in the field of photocatalytic degradation.

**Figure 11:**
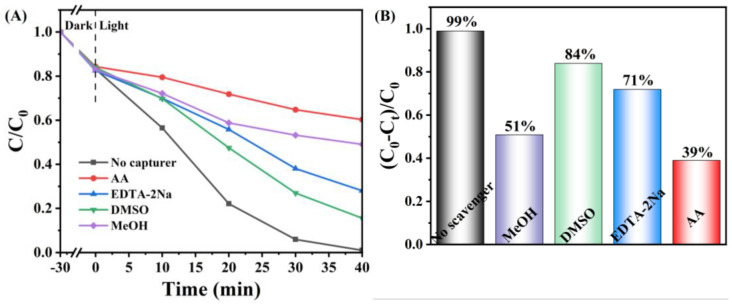
Active species capture assay on the CeO_2_/AgCl-30 composite, (**A**) degradation curves, (**B**) degradation rate.

The reactive species that played a major role in the degradation process were determined by adding different scavengers. Disodium ethylenediaminetetraacetate, ascorbic acid, methanol, and dimethyl sulfoxide were used to scavenge h^+^, ·O_2_^−^, ·OH and e^−^ [46,47,48,49]. In Figure 11, taking the degradation of RhB as an example, the four substances played different degrees of major roles. Notably, compared with the blank, following the addition of AA, the rate of degradation decreased significantly from 99% to 39%, and in the test with MeOH added, the degradation rate decreased to 51%, demonstrating that ·O_2_^−^ and ·OH were the principal reactive species. Meanwhile, after scavenging h^+^ and e^−^, the degradation rates decreased to 71% and 84%, respectively, suggesting that h^+^ and e^−^ also played secondary roles in the degradation.

**Figure 12:**
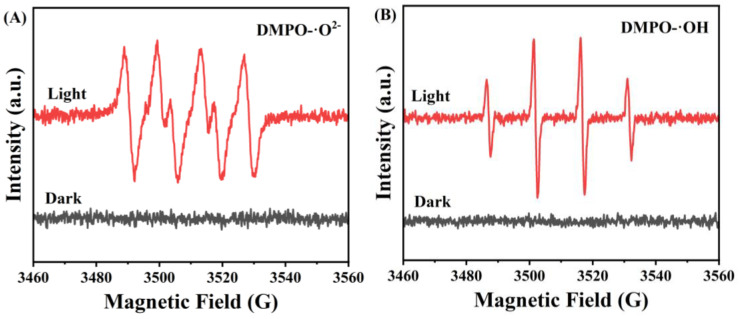
(**A**) DMPO-·O_2_^−^ and (**B**) DMPO-·OH spin-trapping ESR spectroscopy study of the CeO_2_/AgCl-30 composite.

To further verify that large amounts of ·O_2_^−^ and ·OH are evoked amidst the photocatalytic degradation process of the CeO_2_/AgCl, with DMPO as the trapping agent, the CeO_2_/AgCl-30 composite was selected as an instance for ESR testing. It can be observed in Figure 12A,B that, under dark reaction conditions, no signal peaks indicating the generation of free radicals were detected. However, after ten minutes of illumination, distinct signal peaks of ·O_2_^−^ and ·OH were detected. This confirms that the CeO_2_/AgCl composite can generate a large number of ·O_2_^−^ and ·OH under illumination conditions [50,51].

Since the activity of photocatalysts is related to their semiconductor type, conduction band (CB) and valence band (VB), the Mott–Schottky measurement method can be used to determine their band potentials and semiconductor types. Figure S5 shows the Mott–Schottky plots of the pure AgCl as well as the CeO_2_ measured at frequencies of 1000, 1500, and 2000 Hz, respectively. The slopes of the tangents are all positive, indicating that both AgCl and CeO_2_ belong to the n-type semiconductors [52]. The intercepts of the Mott–Schottky curves with the abscissa are E_FB_. The E_FB_ values of AgCl and CeO_2_ relative to the Ag/AgCl electrode are −0.21 V_SCE_ and −1.59 V_SCE_, respectively. For n-type semiconductors, the CB is 0.1 V more negative than the E_FB_. Using the formula E_NHE_ = E_Ag/AgCl_ + 0.198 V [53], the E_CB_ values relative to the normal hydrogen electrode (NHE) are −0.112 V_NHE_ and −1.492 V_NHE_. In the equation E_VB_ = E_CB_ + Eg [54], the VB potentials of AgCl and CeO_2_ are 3.138 VNHE and 1.658 V_NHE_, respectively.

**Figure 13:**
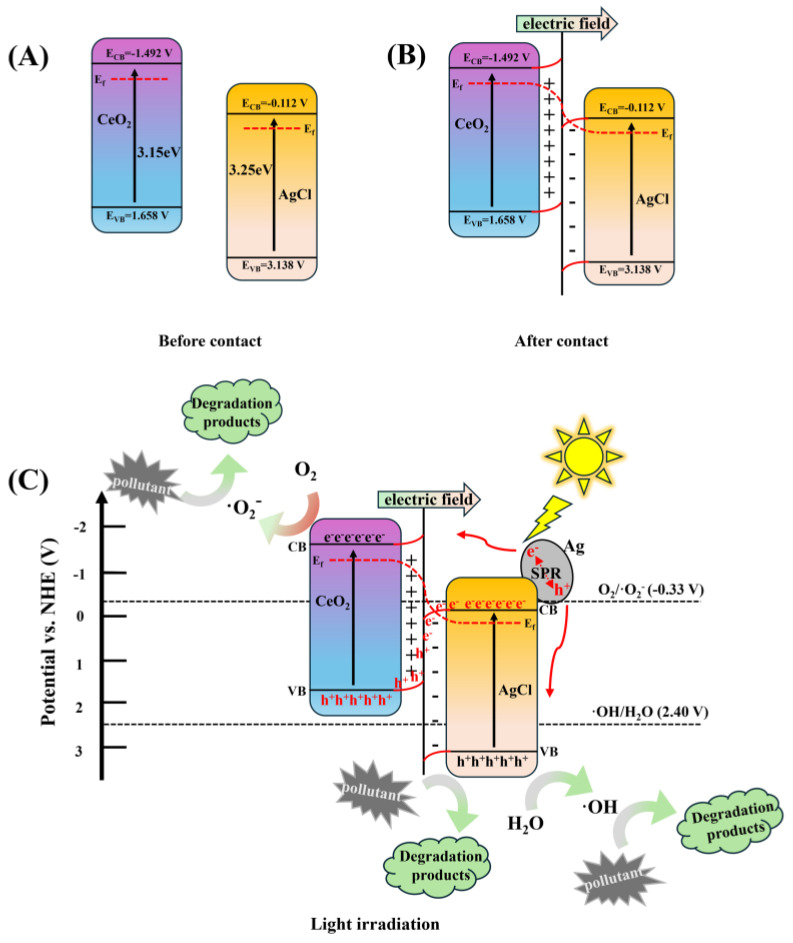
(**A**) The band edge of CeO_2_ and AgCl before contact, (**B**) the internal electric field and band edge bending at the interface of CeO_2_/AgCl after contact, and (**C**) the S-scheme charge transfer mechanism between CeO_2_ and AgCl under light irradiation.

The electron band structure of CeO_2_ prior to its contact with AgCl is depicted in Figure 13A. Generally, for the S-scheme heterojunction formation, the Fermi level (E_f_) and the conduction band (CB) of the reducing semiconductor CeO_2_ are positioned higher than those of the oxidizing semiconductor AgCl. Once in contact, because of the distinct electron density distributions within the bulk phases and at the interfaces of the CeO_2_ and AgCl, the E_f_ in the CeO_2_ and AgCl diverge (Figure 13B). At the AgCl interface, an augmentation of the electron density results in an elevation of the E_f_, while at the CeO_2_ interface, a decline in the electron density causes a reduction in the E_f_. Consequently, the band edges of CeO_2_ and AgCl bend separately in the interfacial zone, thereby creating a built-in electric field [55]. As illustrated in Figure 13C, when the incident light energy exceeds the bandgap of both CeO_2_ and AgCl, electrons in the valence band (VB) are photoexcited to the conduction band (CB), respectively. The valence band potential of CeO_2_ is 1.658 V, which is more negative compared to the potential of the ·OH/H_2_O couple (2.40 V), making it challenging to generate ·OH [56]. The valence band potential of AgCl is 3.138 V, significantly higher than the potential of the ·OH/H_2_O couple (2.40 V). Thus, H_2_O can be oxidized to ·OH by the photogenerated holes (h^+^) in the valence band of AgCl, and ·OH can efficiently decompose RhB. Nevertheless, the conduction band potential of AgCl is −0.112 V, which is higher than the potential of the O_2_/·O_2_^−^ couple (−0.33 V). As a result, the O_2_ in the reaction system cannot be reduced to ·O_2_^−^ by the electrons (e^−^) photoexcited in the conduction band of AgCl, which severely impedes the photocatalytic activity of AgCl. Therefore, the photocatalytic degradation efficiency of either pure CeO_2_ or AgCl for RhB is quite low. After the establishment of the S-scheme heterojunction between CeO_2_ and AgCl, the photogenerated electrons in the conduction band of AgCl recombine with the holes in the valence band of CeO_2_ at the interface, preserving the e^−^ (CeO_2_) and h^+^ (AgCl) with robust redox capabilities. CeO_2_ has a relatively negative conduction band potential (−1.492 V), so the e^−^ in the CB of CeO_2_ can reduce the adsorbed O_2_ to generate ·O_2_^−^. AgCl has a relatively positive VB potential (+3.138 V), enabling the h^+^ with strong oxidation ability remaining in the VB of AgCl to oxidize H_2_O to produce ·OH. Both ·O_2_^−^ and ·OH can facilitate the decomposition of RhB and the h^+^ in the valence band of AgCl can also directly degrade RhB molecules [57]. In addition, due to the photodissociation of AgCl, silver is formed on the surface. Silver nanoparticles can be excited through the SPR effect, thereby generating electrons and holes [58]. On the one hand, the excited electrons can be transferred to the conduction band of CeO_2_, promoting the generation of superoxide radicals. On the other hand, the holes formed on Ag NPs can also be transferred to the surface of AgCl.

Through the above mechanisms, not only are electrons and holes effectively separated in the composite material but also more reactive oxygen species can be formed. This is beneficial for improving the photocatalytic activity of the CeO_2_/AgCl composite material.

**Figure 14:**
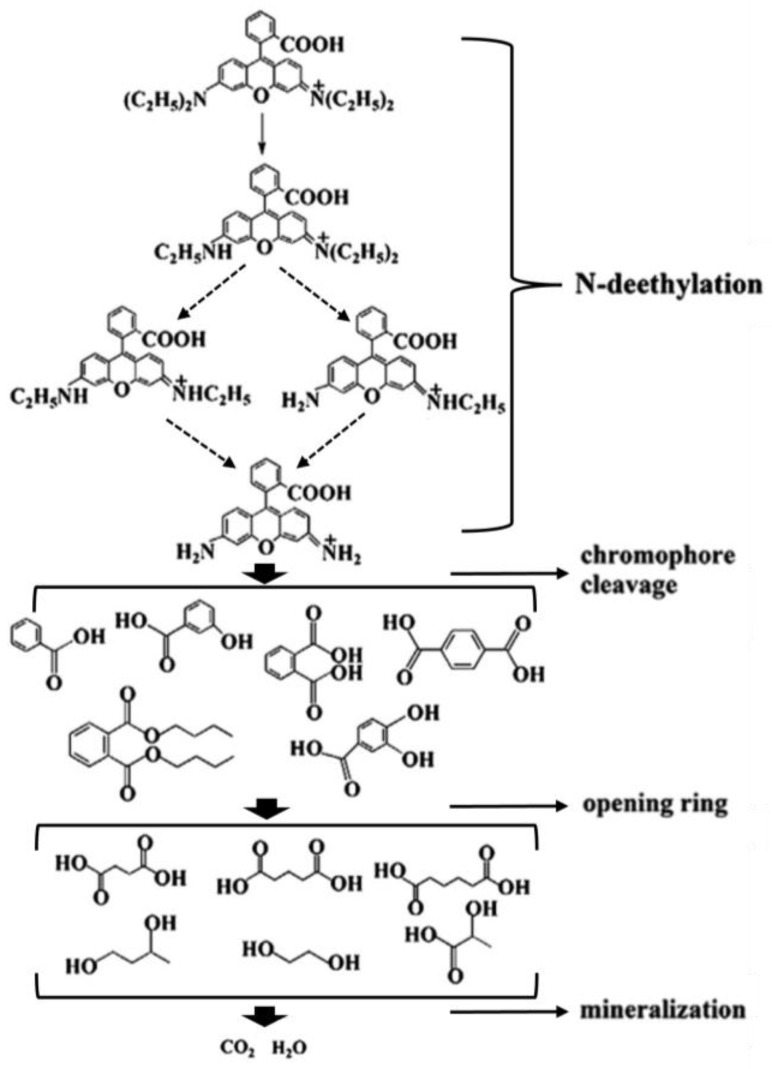
The possible degradation intermediate and the final products of RhB over CeO_2_/AgCl-30.

Finally, Figure 14 shows the possible degradation intermediate products and final products of RhB on the composite material CeO_2_/AgCl-30 during the photocatalysis process. According to the previous literature [4,59], the photocatalytic degradation process of RhB mainly includes four steps, namely N-demethylation, chromophore cleavage, opening ring, and mineralization. The final degradation products are CO_2_ and H_2_O. In our photocatalytic degradation process, no obvious organic compounds were detected either, indicating that the final degradation products of RhB are carbon dioxide and water.

## 4. Conclusions

A novel CeO_2_/AgCl photocatalyst was successfully synthesized through a one-step chemical precipitation technique. By combining CeO_2_ with AgCl to construct an S-scheme heterojunction, the recombination of photogenerated charges and holes can be strongly restrained, reducing the charge transfer resistance, and the photocatalytic ability can be enhanced. The photocatalytic degradation exhibited by the CeO_2_/AgCl composite of various organic pollutants is superior to that of pure AgCl. The highest degradation rate can reach 99% within 40 min, and it is not affected by environmental changes. Meanwhile, experiments have proven that ·O_2_^−^ and ·OH are the key reactive species, and they can efficiently decompose organic dyes and ultimately obtain small molecules that are harmful to the environment. The photocatalytic stability was also demonstrated in four cyclic experiments and via XRD, FTIR, SEM analysis, which is of great significance for the practical application of the materials. These results demonstrate the application potential of CeO_2_/AgCl in water treatment, clarify the preparation of highly efficient photocatalysts, and may solve the problem of pollutant degradation. This broadens the application scope of photocatalytic technology in the field of environmental remediation. Secondly, the deeper understanding of the interaction mechanism between CeO_2_ and AgCl, as well as the photocatalytic degradation mechanism of RhB, has enriched the theoretical knowledge in the field of photocatalysis.

## Data Availability

Data is contained within the article or Supplementary Materials.

## Supplementary Materials

The following supporting information can be downloaded at: https://www.mdpi.com/article/10.3390/nano15070537/s1, Figure S1. FTIR spectra of AgCl, CeO_2_/AgCl-10, CeO_2_/AgCl-30, CeO_2_/AgCl-50 and CeO_2_. Figure S2. EDS elemental mapping of Ag, Cl, O and Ce and EDS spectrum of CeO₂/AgCl-30. Figure S3. (A) UV-vis DRS over CeO_2_, (B) band gap of CeO_2_ and (C) band gap of CeO_2_/AgCl-30. Figure S4. Equivalent circuit for EIS. Table S1. Fitted Results of EIS Spectra. Figure S5. Mott-Schottky tangent plots of (A) AgCl and (B) CeO_2_.

## References

[1] Chong M.N., Jin B., Chow C.W.K., Saint C. (2010). Recent developments in photocatalytic water treatment technology: A review. Water Res..

[2] Long Z., Li Q., Wei T., Zhang G., Ren Z. (2020). Historical development and prospects of photocatalysts for pollutant removal in water. J. Hazard. Mater..

[3] Li Q., Li F. (2021). Recent advances in molecular oxygen activation via photocatalysis and its application in oxidation reactions. Chem. Eng. J..

[4] Shi L., Ma J., Yao L.Z., Cui L.S., Qi W. (2018). Enhanced photocatalytic activity of Bi_12_O_17_Cl_2_ nano-sheets via surface modification of carbon nanotubes as electron carriers. J. Colloid. Interf. Sci..

[5] Wang Y., Liu H., Wu B., Zhou T., Wang J., Zhou J., Li S., Cao F., Qin G. (2019). Preparation and visible-light-driven photocatalytic property of AgX (X= Cl, Br, I) nanomaterials. J. Alloy Compd..

[6] An C., Wang S., Sun Y., Zhang Q., Zhang J., Wang C., Fang J. (2016). Plasmonic silver incorporated silver halides for efficient photocatalysis. J. Mater. Chem. A.

[7] Zhao X., Lv X., Cui H., Wang T. (2017). Preparation of bismuth stannate/silver@ silver chloride film samples with enhanced photocatalytic performance and self-cleaning ability. J. Colloid. Interf. Sci..

[8] Weng B., Qi M.Y., Han C., Tang Z.R., Xu Y.J. (2019). Photocorrosion inhibition of semiconductor-based photocatalysts: Basic principle, current development, and future perspective. ACS Catal..

[9] Yu J., Sun D., Wang T., Li F. (2018). Fabrication of Ag@ AgCl/ZnO submicron wire film catalyst on glass substrate with excellent visible light photocatalytic activity and reusability. Chem. Eng. J..

[10] Mohseni N., Haghighi M., Shabani M. (2022). Bimetallic Co^2+^ Cr^3+^ LDH anchored AgCl as excellent solar-light-responsive Ag-decorated type II nano-heterojunction for photodegradation of various dyes. Process Saf. Environ..

[11] Sun C., Li H., Chen L. (2012). Nanostructured ceria-based materials: Synthesis, properties, and applications. Energ. Environ. Sci..

[12] Das H.T., Dutta S., Das N., Das P., Mondal A., Imran M. (2022). Recent trend of CeO_2_-based nanocomposites electrode in supercapacitor: A review on energy storage applications. J. Energy Storage.

[13] Trovarelli A. (1996). Catalytic properties of ceria and CeO_2_-containing materials. Catal. Rev..

[14] Kim J.Y., Jang B., Lim M., Park J.Y., Choa Y.H. (2024). Enhanced PtRu by CeO_2_ hollow nanofibers: Hydrogen gas sensing with CO-resistant in fuel cell. J. Power Sources.

[15] Agarwal U.P., Ralph S.A., Baez C., Reiner R.S., Verrill S.P. (2017). Effect of sample moisture content on XRD-estimated cellulose crystallinity index and crystallite size. Cellulose.

[16] Yin H., Wang X., Wang L., Nie Q., Zhang Y., Yuan Q., Wu W. (2016). Ag/AgCl modified self-doped TiO_2_ hollow sphere with enhanced visible light photocatalytic activity. J. Alloy Compd..

[17] Wang P., Huang B., Lou Z., Zhang X., Qin X., Dai Y., Zheng Z., Wang X. (2010). Synthesis of highly efficient Ag@ AgCl plasmonic photocatalysts with various structures. Chem-Asianj.

[18] Danish M., Sandhu Z.A., Sharif S., Raza M.A., Elahi M., Aslam M., Zain M., Batoo K.M., Ijaz M.F. (2024). Excellence of Engineered Ce_2_O_3_ and Bi_2_O_3_@ Ce_2_O_3_ Nanomaterials for Unveiling the Pseudocapacitive and Catalytic Applications. J. Inorg. Organomet P.

[19] Qiao R., Mao M., Hu E., Zhong Y., Ning J., Hu Y. (2015). Facile formation of mesoporous BiVO_4_/Ag/AgCl heterostructured microspheres with enhanced visible-light photoactivity. Inorg. Chem..

[20] Brant P., Stephenson T.A. (1987). X-ray photoelectron spectra of mixed-valence diruthenium complexes. Distinguishing between strongly and weakly interacting metals in delocalized class III complexes. Inorg. Chem..

[21] Alonso J.A. (2000). Electronic and atomic structure, and magnetism of transition-metal clusters. Chem. Rev..

[22] Winter B., Faubel M. (2006). Photoemission from liquid aqueous solutions. Chem. Rev..

[23] Jaffari G.H., Imran A., Bah M., Ali A., Bhatti A.S., Qurashi U.S., Shah S.I. (2017). Identification and quantification of oxygen vacancies in CeO_2_ nanocrystals and their role in formation of F-centers. Appl. Surf. Sci..

[24] Li X., Yao C., Lu X., Hu Z., Yin Y., Ni C. (2015). Halloysite–CeO_2_–AgBr nanocomposite for solar light photodegradation of methyl orange. Appl. Clay Sci..

[25] Su F., Li P., Huang J., Gu M., Liu Z., Xu Y. (2021). Photocatalytic degradation of organic dye and tetracycline by ternary Ag_2_O/AgBr–CeO_2_ photocatalyst under visible-light irradiation. Sci. Rep-UK.

[26] Lin X., Liu H., Guo B., Zhang X. (2016). Synthesis of AgCl/Ag/AgCl core-shell microstructures with enhanced photocatalytic activity under sunlight irradiation. J. Environ. Chem. Eng..

[27] Han S.H., Liu H.M., Sun C.C., Jin P.J., Chen U. (2018). Photocatalytic performance of AgCl@ Ag core–shell nanocubes for the hexavalent chromium reduction. J. Mater. Sci..

[28] Shi L., Huang Y., Seo H.J. (2010). Emission red shift and unusual band narrowing of Mn^2+^ in NaCaPO_4_ phosphor. J. Phys. Chem. A.

[29] Haddad R.E., Gazeau S., Pécaut J., Marchon J.C., Medforth C.J., Shelnutt J.A. (2003). Origin of the red shifts in the optical absorption bands of nonplanar tetraalkylporphyrins. J. Am. Chem. Soc..

[30] Dai W., Hu X., Wang T., Xiong W., Luo X., Zou J. (2018). Hierarchical CeO_2_/Bi_2_MoO_6_ heterostructured nanocomposites for photoreduction of CO_2_ into hydrocarbons under visible light irradiation. Appl. Surf. Sci..

[31] Jakimińska A., Macyk W. (2023). Photochemical transformations of AgCl in the context of its eventual photocatalytic applications. J. Photoch Photobio A.

[32] Fan Y., Bao Y., Song Z., Sun Z., Wang D., Han D., Niu L. (2018). Controllable synthesis of coloured Ag 0/AgCl with spectral analysis for photocatalysis. Rsc. Adv..

[33] Wang X., Li S., Ma Y., Yu H., Yu J. (2011). H_2_WO_4_·3H_2_O/Ag/AgCl composite nanoplates: A plasmonic Z-scheme visible-light photocatalyst. J. Phys. Chem. C.

[34] Chen D., Li T., Chen Q., Gao J., Fan B., Li J., Li X., Zhang R., Sun J., Gao L. (2012). Hierarchically plasmonic photocatalysts of Ag/AgCl nanocrystals coupled with single-crystalline WO_3_ nanoplates. Nanoscale.

[35] Myrick M.L., Simcock M.N., Baranowski M., Brooke H., Morgan S.L., McCutcheon J.N. (2011). The Kubelka-Munk diffuse reflectance formula revisited. Appl. Spectrosc. Rev..

[36] Landi S., Segundo I.R., Freitas E., Vasilevskiy M., Carneiro J., Tavares C.J. (2022). Use and misuse of the Kubelka-Munk function to obtain the band gap energy from diffuse reflectance measurements. Solid. State Commun..

[37] Han C., Ge L., Chen C., Li Y., Zhao Z., Xiao X., Li Z., Zhang J. (2014). Site-selected synthesis of novel Ag@ AgCl nanoframes with efficient visible light induced photocatalytic activity. J. Mater. Chem. A.

[38] Majher J.D., Gray M.B., Strom T.A., Woodward P.M. (2019). Cs_2_NaBiCl_6_: Mn^2+^—A new orange-red halide double perovskite phosphor. Chem. Mater..

[39] Low J., Yu J., Jaroniec M., Wageh S., Al-Ghamdi A.A. (2017). Heterojunction photocatalysts. Adv. Mater..

[40] Yang H. (2021). A short review on heterojunction photocatalysts: Carrier transfer behavior and photocatalytic mechanisms. Mater. Res. Bull..

[41] Bi L., Gao X., Zhang L., Wang D., Zou X., Xie T. (2018). Enhanced photocatalytic hydrogen evolution of NiCoP/g-C_3_N_4_ with improved separation efficiency and charge transfer efficiency. ChemSusChem.

[42] Xu H., Chang Y., Shen X., Liu Z., Zhu B., Macharia D.K., Wang Z., Chen Z., Zhang L. (2019). Construction of Ag/AgCl-CN heterojunctions with enhanced photocatalytic activities for degrading contaminants in wastewater. J. Colloid. Interf. Sci..

[43] Zhao K., Zhang Z., Feng Y., Lin S., Li H., Gao X. (2020). Surface oxygen vacancy modified Bi_2_MoO_6_/MIL-88B (Fe) heterostructure with enhanced spatial charge separation at the bulk & interface. Appl. Catal. B-Environ..

[44] Yu S., Li J., Zhang Y., Li M., Dong F., Zhang T., Huang H. (2018). Local spatial charge separation and proton activation induced by surface hydroxylation promoting photocatalytic hydrogen evolution of polymeric carbon nitride. Nano Energy.

[45] Simonin J.P. (2016). On the comparison of pseudo-first order and pseudo-second order rate laws in the modeling of adsorption kinetics. Chem. Eng. J..

[46] Zhang X., Dou J., Yan S., Yao L., Fu Y., Shi L. (2021). Enhanced Photocatalytic Activity of AgI/BiO_2_-x Heterojunction Photocatalyst. ChemistrySelect.

[47] He C., Jing P., Wang P., Ji J., Ouyang T., Cui Y., Pu Y. (2021). A novel hierarchical BaTiO_3_/AgI heterojunction with boosting spatial charge kinetics for photocatalytic degradation of organic pollutant. Ceram. Int..

[48] Wang F., Zhou F., Zhan S., He Q., Song Y., Zhang C., Lai J. (2021). Morphology modulation and performance optimization of nanopetal-based Ag-modified Bi_2_O_2_CO_3_ as an inactivating photocatalytic material. Environ. Res..

[49] Demeestere K., Dewulf J., De Witte B., Van Langenhove H. (2005). Titanium dioxide mediated heterogeneous photocatalytic degradation of gaseous dimethyl sulfide: Parameter study and reaction pathways. Appl. Catal. B-Environ..

[50] Chang C.R., Wang Y.G., Li J. (2011). Theoretical investigations of the catalytic role of water in propene epoxidation on gold nanoclusters: A hydroperoxyl-mediated pathway. Nano Res..

[51] Liu L.M., Crawford P., Hu P. (2009). The interaction between adsorbed OH and O_2_ on TiO_2_ surfaces. Prog. Surf. Sci..

[52] Padma R., Sreenu K., Reddy V.R. (2017). Electrical and frequency dependence characteristics of Ti/polyethylene oxide (PEO)/p-type InP organic-inorganic Schottky junction. J. Alloy Compd..

[53] Lee M.H., Takei K., Zhang J., Kapadia R., Zheng M., Chen Y.Z., Nah J., Matthews T., Chueh Y.L., Ager J. (2012). p-Type InP nanopillar photocathodes for efficient solar-driven hydrogen production. Angew. Chem. Int. Edit.

[54] Wang J., Zhang J., Peh S.B., Liu G., Kundu T., Dong J., Ying Y., Qian Y., Zhao D. (2020). Cobalt-containing covalent organic frameworks for visible light-driven hydrogen evolution. Sci. China Chem..

[55] Xu Q., Wageh S., Al-Ghamdi A.A., Li X. (2022). Design principle of S-scheme heterojunction photocatalyst. J. Mater. Sci. Technol..

[56] Zhang X., Zhang H., Yu J., Wu Z., Zhou Q. (2022). Preparation of flower-like Co_3_O_4_ QDs/ Bi_2_WO_6_ p-n heterojunction photocatalyst and its degradation mechanism of efficient visible-light-driven photocatalytic tetracycline antibiotics. Appl. Surf. Sci..

[57] Quan Y., Liu M., Wu H., Tian X., Dou L., Wang Z., Ren C. (2024). Rational design and construction of S-scheme CeO_2_/AgCl heterojunction with enhanced photocatalytic performance for tetracycline degradation. Appl. Surf. Sci..

[58] Pal A., Jana A., Bhattacharya S., Datta J. (2017). SPR effect of AgNPs decorated TiO_2_ in DSSC using TPMPI in the electrolyte: Approach towards low light trapping. Electrochim. Acta.

[59] He Z., Sun C., Yang S., Ding Y., He H., Wang Z. (2009). Photocatalytic degradation of rhodamine B by Bi_2_WO_6_ with electron accepting agent under microwave irradiation: Mechanism and pathway. J. Hazard. Mater..

