# Supplementary material for: Enhancing Visible-Light Photocatalytic Activity of AgCl Photocatalyst by CeO2 Modification for Degrading Multiple Organic Pollutants
Source: Nanomaterials (Basel). 2025 Apr 1;15(7):537. doi: 10.3390/nano15070537 (PMC11990855; doi:10.3390/nano15070537)
Supplement: Supplementary file 1 [file nanomaterials-15-00537-s001.zip › nanomaterials-3523329-supplementary.pdf]

Enhancing Visible-Light Photocatalytic Activity of AgCl Photocatalyst by CeO_2 Modification for Degrading Multiple Organic Pollutants

Li Xu [1,*], Ning Yang [1], Tong Xu [1,2,*], Yang Yang [2] and Yanfei Lv [2]

[1] *Novel Energy Materials & Catalysis Research Center, Shanwei Innovation Industrial Design & Research Institute, Shanwei 516600, China*

[2] *Laboratory of Plasma Catalysis, Dalian Maritime University, Dalian 116026, China*

* *Corresponding authors. E-mail addresses: xuli3021@gmail.com (L. Xu), heyangxv@dlmu.edu.cn (T. Xu)*

Characterization of catalyst

The X-ray diffraction (XRD) was detected on a Japanese Institute of Technology Smartlab SE. FTIR were detected on Thermo Nicolet iS5. Thermo Fisher ESCALAB 250Xi was used to measure X-ray photoelectron spectroscopy (XPS). The surface feature was observed by Hitachi scanning electron microscope (SEM, SU8600), and elements mapping images were measured by Oxford Multimax 40. Photoresponsiveness was recorded using an Agilent Cary 5000 UV-Vis diffuse reflectance spectroscopy (DRS). Photoluminescence spectra (PL) were recorded using an Edinburgh FLS-1000. Use Brooke A300 to test electron spin resonance (ESR). Photocurrent, electrochemical impedance spectra (EIS) and Mott-Schottky curves were recorded on a Chenhua electrochemical station (CHI660E) in a three electrodes system. The total organic carbon (TOC) content for the dye degradation was analyzed on vario TOC cube (Elementar Analysensysteme GmbH).

Photocatalytic degradation test

Rhodamine B (RhB) as a model pollutant was used in photocatalytic degradation experiments. A 300 W Xe lamp with 420 nm cut-off filter was applied as the visible-light source. 30 mg photocatalyst sample was added into 50 mL 10 mg/L RhB solution and stirred for an hour in the dark to reach adsorption-desorption equilibrium. During photocatalytic degradation reaction, 4 mL solution was taken every 10 min and centrifuged (8000 rpm, 5 min) twice. The gained solution was measured at 553 nm on UV-vis spectrometer.

The degradation steps of methyl orange (MO) and crystal violet (CV) are the same as those of RhB. The gained solution was measured at 464 nm and 588 nm on UV-vis spectrometer,

respectively.

Cycle test for the photocatalyst

A recovery test for the degradation of RhB was carried out with CeO_2/AgCl-30. The first cycle is the same as the "photocatalytic degradation test" procedure described above. For the second cycle, the photocatalyst was subjected to a full photocatalytic degradation process. The photodegradation of 50 ml of a 10 mg/L RhB solution was performed with 30 mg of CeO_2/AgCl-30 nanocomposite under visible light irradiation. After eliminating the color of the solution, the solution was removed Fresh 50 mL of the 10 mg/L RhB solution was then added to the reactor and the degradation data was collected according to the "Photocatalytic Degradation Test" procedure. In the third cycle, the photocatalyst was subjected to the entire photocatalytic degradation process twice. Other steps are the same as in the second cycle. In the fourth cycle, the photocatalyst was subjected to the entire photocatalytic degradation process Three times. Other steps are the same as in the second cycle.

Detection of reactive species

This experimental process was just like photocatalytic degradation test, the only difference was that the quencher was added to the RhB solution together with the photocatalyst. The quenchers included disodium ethylene diamine tetraacetate (EDTA-2Na, 10 mg), methyl alcohol (2 mL), ascorbic acid (10 mg) and dimethyl sulfoxide (2 mL).

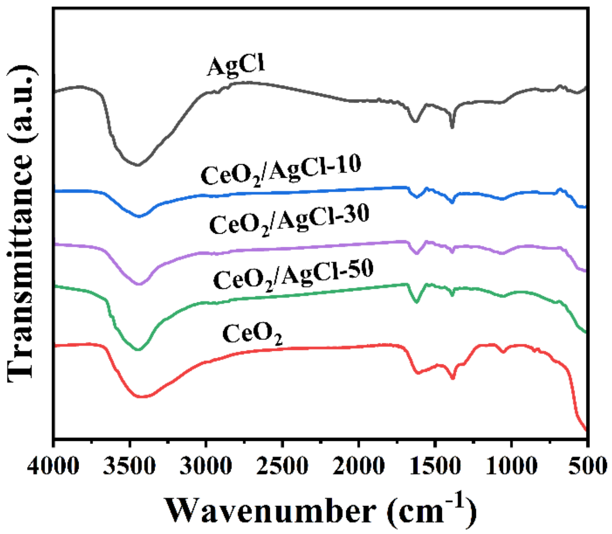

Figure S1. FTIR spectra of AgCl, CeO_2/AgCl-10, CeO_2/AgCl-30, CeO_2/AgCl-50 and CeO_2.

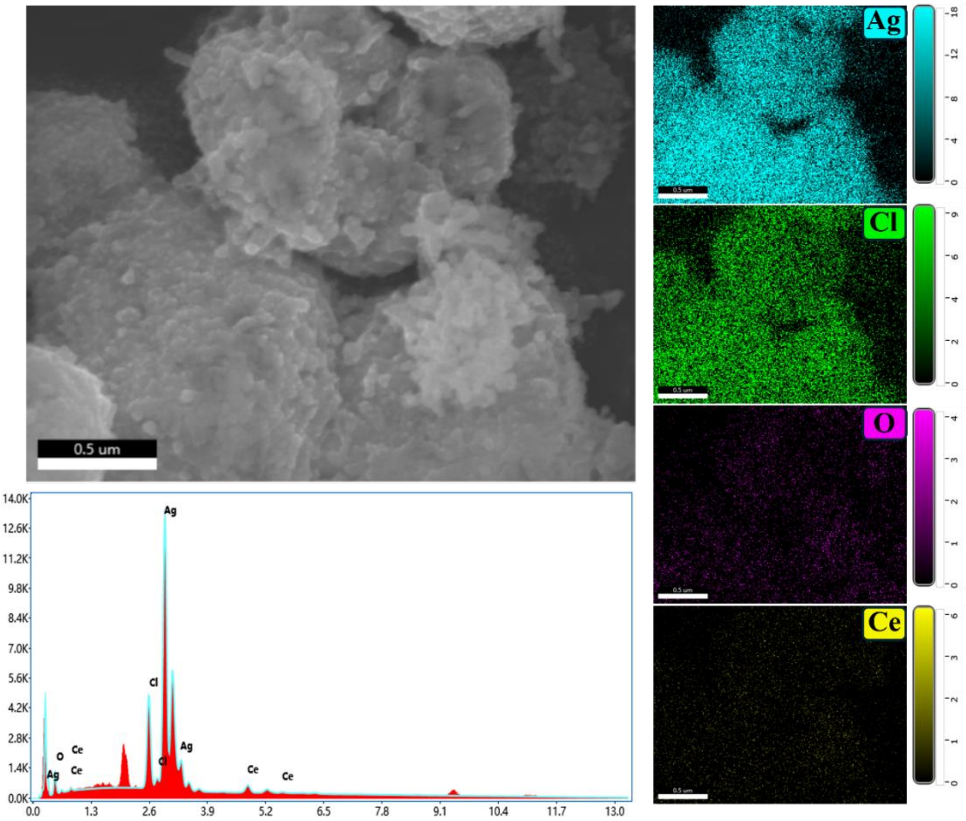

Figure S2. EDS elemental mapping of Ag, Cl, O and Ce and EDS spectrum of CeO_2/AgCl-30.

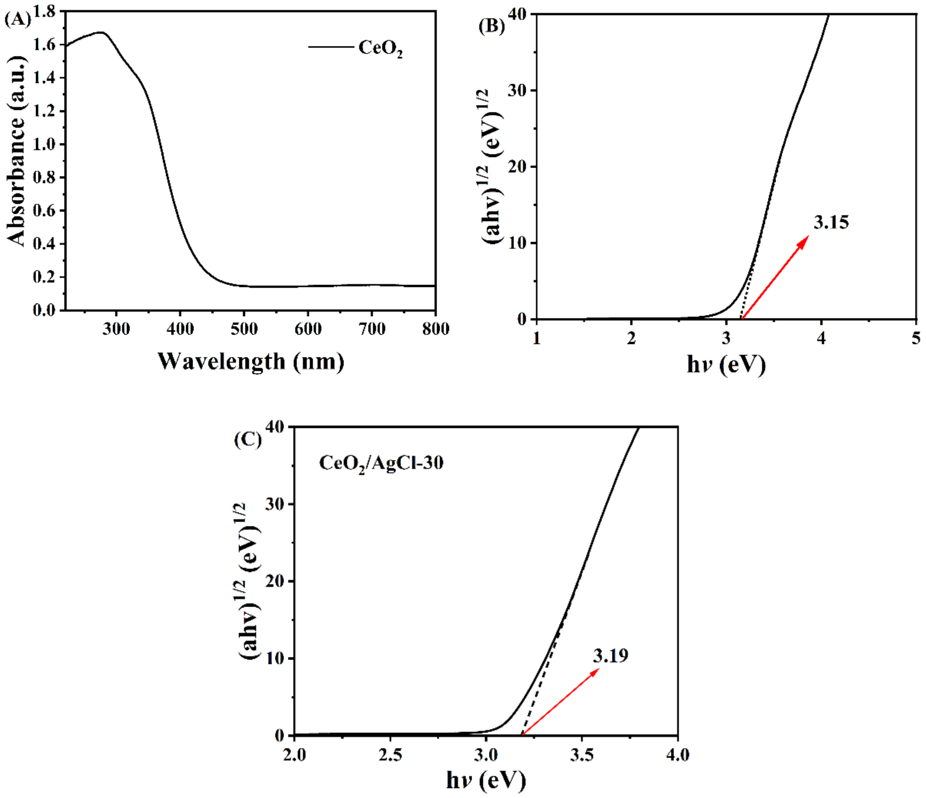

Figure S3. (A) UV-vis DRS over CeO_2, (B) band gap of CeO_2 and (C) band gap of CeO_2/AgCl-30

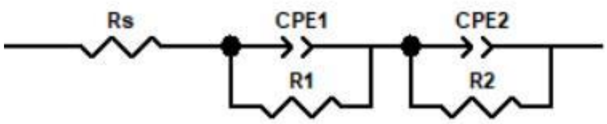

Figure S4. Equivalent circuit for EIS.

Table S1. Fitted Results of EIS Spectra

parameters	Rs (kΩ)	CPE1-T (×10^{-6} F)	CPE1-P	R1 (kΩ)	CPE2-T (×10^{-6} F)	CPE2-P	R2 (kΩ)
CeO_2/AgCl-30	0.106	0.711	0.374	0.118	205.89	10.29	0.0102
AgCl	0.166	1.315	0.445	0.182	185.94	16.12	0.0161

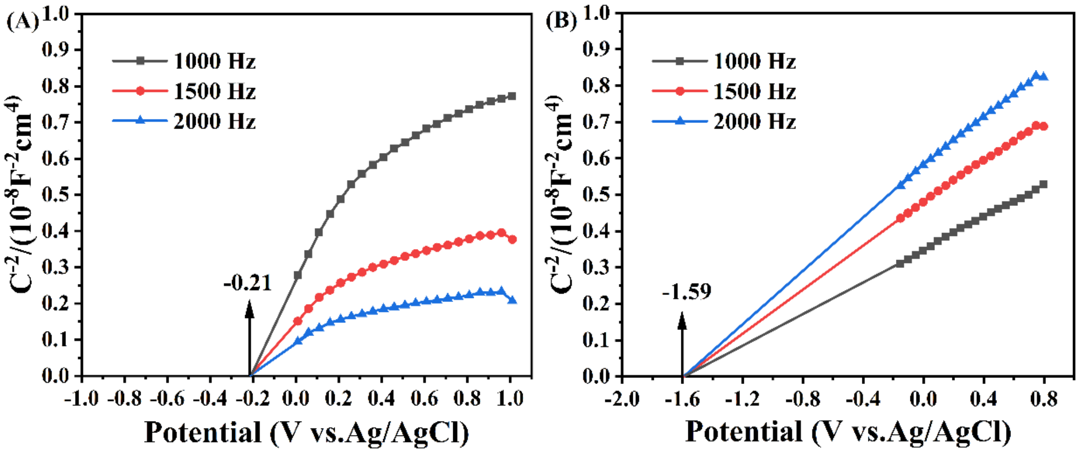

Figure S5. Mott - Schottky tangent plots of (A) AgCl and (B) CeO_2.
